# Extracellular vesicles released from ganglioside GD2-expressing melanoma cells enhance the malignant properties of GD2-negative melanomas

**DOI:** 10.1038/s41598-023-31216-4

**Published:** 2023-03-27

**Authors:** Farhana Yesmin, Keiko Furukawa, Mariko Kambe, Yuhsuke Ohmi, Robiul Hasan Bhuiyan, Mohammad Abul Hasnat, Momoka Mizutani, Orie Tajima, Noboru Hashimoto, Akiko Tsuchida, Kei Kaneko, Koichi Furukawa

**Affiliations:** 1 Department of Biomedical Sciences, Chubu University College of Life and Health Sciences, Matsumoto 1200, Kasugai, Aichi 487-8501 Japan; 2 Department of Clinical Engineering, Chubu University College of Life and Health Sciences, Matsumoto 1200, Kasugai, Aichi 487-8501 Japan; 3 Department of Tissue Regeneration, Tokushima University School of Dentistry, Kuramoto-Cho 3, Tokushima, 770-8504 Japan; 4 Laboratory of Glyco- Bioengineering, The Noguchi Institute, Itabashi, 173-0003 Japan; 5 Department of Biochemistry and Molecular Biology, University of Chittagong, 4331 Chittagong, Bangladesh

**Keywords:** Glycoconjugates, Tumour immunology, Immunosurveillance, Immunoediting, Cancer, Biochemistry, Carbohydrates, Glycobiology

## Abstract

Exosomes (small extracellular vesicles: EVs) have attracted increasing attention from basic scientists and clinicians since they play important roles in cell-to-cell communication in various biological processes. Various features of EVs have been elucidated regarding their contents, generation and secretion mechanisms, and functions in inflammation, regeneration, and cancers. These vesicles are reported to contain proteins, RNAs, microRNAs, DNAs, and lipids. Although the roles of individual components have been rigorously studied, the presence and roles of glycans in EVs have rarely been reported. In particular, glycosphingolipids in EVs have not been investigated to date. In this study, the expression and function of a representative cancer-associated ganglioside, GD2, in malignant melanomas was investigated. Generally, cancer-associated gangliosides have been shown to enhance malignant properties and signals in cancers. Notably, EVs derived from GD2-expressing melanomas enhanced the malignant phenotypes of GD2-negative melanomas, such as cell growth, invasion, and cell adhesion, in a dose-dependent manner. The EVs also induced increased phosphorylation of signaling molecules such as EGF receptor and focal adhesion kinase. These results suggest that EVs released from cancer-associated ganglioside-expressing cells exert many functions that have been reported as a function of these gangliosides and regulate microenvironments, including total aggravation of heterogeneous cancer tissues, leading to more malignant and advanced cancer types.

## Introduction

Acidic glycosphingolipids, gangliosides, are mainly expressed in the brains and nervous tissues of vertebrates^1,2^. Some of these molecules are considered tumor markers of neuroectoderm-derived cancers such as malignant melanomas^3,4^, neuroblastomas^5^, and gliomas^6–8^. In addition to these tumors, some gangliosides, such as GD3 and GD2, have also been reported to be characteristically expressed in osteosarcomas^9–11^, T-cell leukemias^12–15^, lung cancers^16,17^, and breast cancers^18^. Moreover, the roles of these gangliosides in cancer cells have been revealed, indicating that these gangliosides play important roles in the enhancement of malignant features of cancers such as increased proliferation, invasion^19^ and cell adhesion^20,21^ and a high incidence of metastasis^22^. As a basis for these functions of cancer-associated gangliosides, their roles in the modulation of cell signaling at membrane microdomains, i.e., lipid rafts, have been reported^23,24^. Many of these studies have been achieved through novel genetic engineering of the expression of glycosphingolipids using cDNAs and genomic DNA of cloned glycosyltransferase genes^25^.

Recently, small extracellular vesicles (EVs) released from various cells have attracted the interests of many biologists, since these EVs have been shown to play important roles in cell-to-cell communication^26,27^, although such vesicles were previously considered to be waste-like nonsignificant particles^28^. In particular, EVs from cancer cells have been rigorously studied and demonstrated to be involved in the induction of experimental metastasis of melanomas^29^ and in the formation of metastatic niche at the target organs as an important organotropism-determining factor of distant metastasis^30^. Furthermore, a number of studies have reported the roles of EVs in the regulation of the surrounding normal cells of tumors^31–33^. The microRNAs included in EVs were also reported to be a reliable diagnostic marker for various cancers^34–36^.

Although there are many molecules, including membrane molecules such as MHC, tetraspanins, integrins, DNA, RNA, microRNA, and many other proteins, in EVs^37^, no definite studies on the presence and function of glycosphingolipids on EVs have been performed to date.

Recently, we investigated how cancer-associated gangliosides enhance malignant signals using a novel technique, EMARS/MS (enzyme-mediated activation of radical sources/mass spectrometry) developed by Kotani and Honke^38^. We reported interesting newly defined membrane molecules, e.g., neogenin in melanomas^39^ and PDGF receptor in gliomas^40^. We also identified integrin β1 as a GD2-associating molecule in melanomas by EMARS/MS^41^ and reported that the molecular complex of GD2 and integrin enhanced the malignant properties of melanomas at lipid rafts.

In this study, we investigated the roles of EVs released from GD2-positive melanoma cells in the regulation of malignant properties of advanced melanomas, such as increased cell growth, high invasion and increased cell adhesion, indicating roles of EVs in the generation of the tumor microenvironment and total aggravation of tumor phenotypes. The results obtained in this study suggest that some ganglioside functions in cancers detected in past studies are exerted via EVs released from ganglioside-expressing cancer cells.

## Results

**Figure 1:**
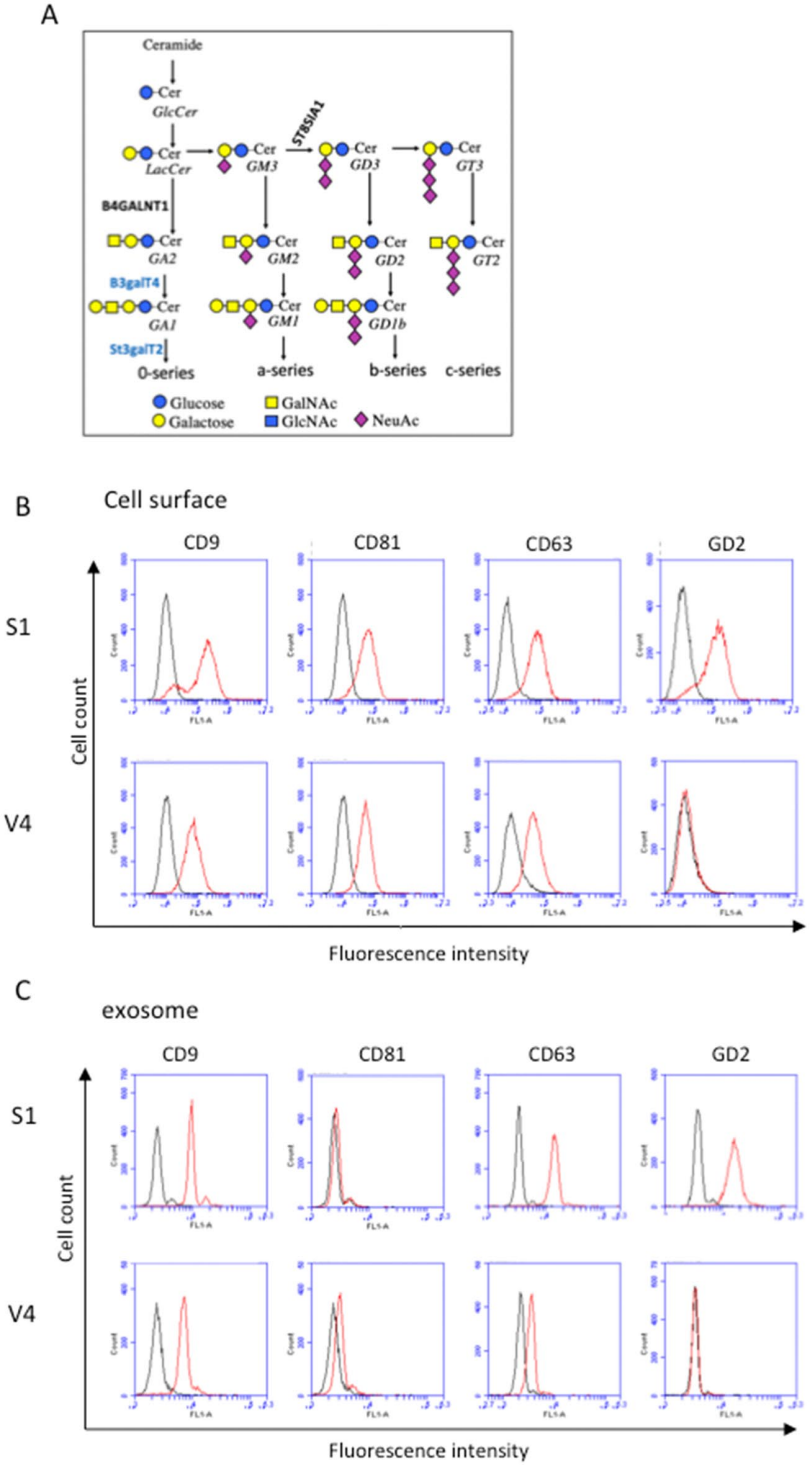
Ganglioside synthetic pathway and expression of tetraspanins and GD2 on the cell surface of transfected cells and on EVs released from them. (**A**) Ganglioside biosynthetic pathway and cDNAs of synthetic enzymes used to establish GD2-expressing transfectant cells. (**B**) Expression of tetraspanins and ganglioside GD2 in GD2+ S1 cells and GD2- V4 cells as analyzed by flow cytometry. Anti-CD9, anti-CD81, anti-CD63 and anti-GD2 mAbs were used as the primary antibodies, and FITC-labeled secondary antibody (goat anti-mouse IgG (H & L) was used. A flow cytometer, AccuriC6, was used. (**C**) Expression of tetraspanins and GD2 on EVs was analyzed using Tim4 beads. EVs were mixed with PS-capture Tim4 beads for 1 h with slight vortexing every 10 min. EV-bound Tim4 beads were washed twice, and primary antibodies against EV markers or GD2 were applied and incubated for 1 h on ice. After washing, secondary antibodies were added and incubated for 1 h. Then, EVs bound to Tim4 beads were washed and applied for flow cytometry as described in (**B**). These results are the representative data of repeated experiments with similar patterns.

Similar expression of tetraspanin on GD2-expressing and GD2-negative melanoma lines, but not on exosomes derived from them. Melanoma subline N1 was transfected with cDNAs of GD3 synthase and GM2/GD2 synthase (Fig. 1A), and GD2-expressing S1 cells were established^41^. Vector controls with no GD2 expression, e.g., V4 cells were also established (Fig. 1B). The expression of tetraspanins was also compared between these two groups, showing no clear differences in CD9 and CD81 by flow cytometry (Fig. 1B). For CD63, S1 cells showed higher levels than V4 cells (Fig. 1B). When EVs were analyzed for the expression of gangliosides and tetraspanins by flow cytometry using Tim4 beads, some differences were detected in CD9 and CD63 (Fig. 1C). Generally, the expression of ganglioside and tetraspanins was similar between the cell surface and surface of EVs, except for CD81 and CD63.

**Figure 2:**
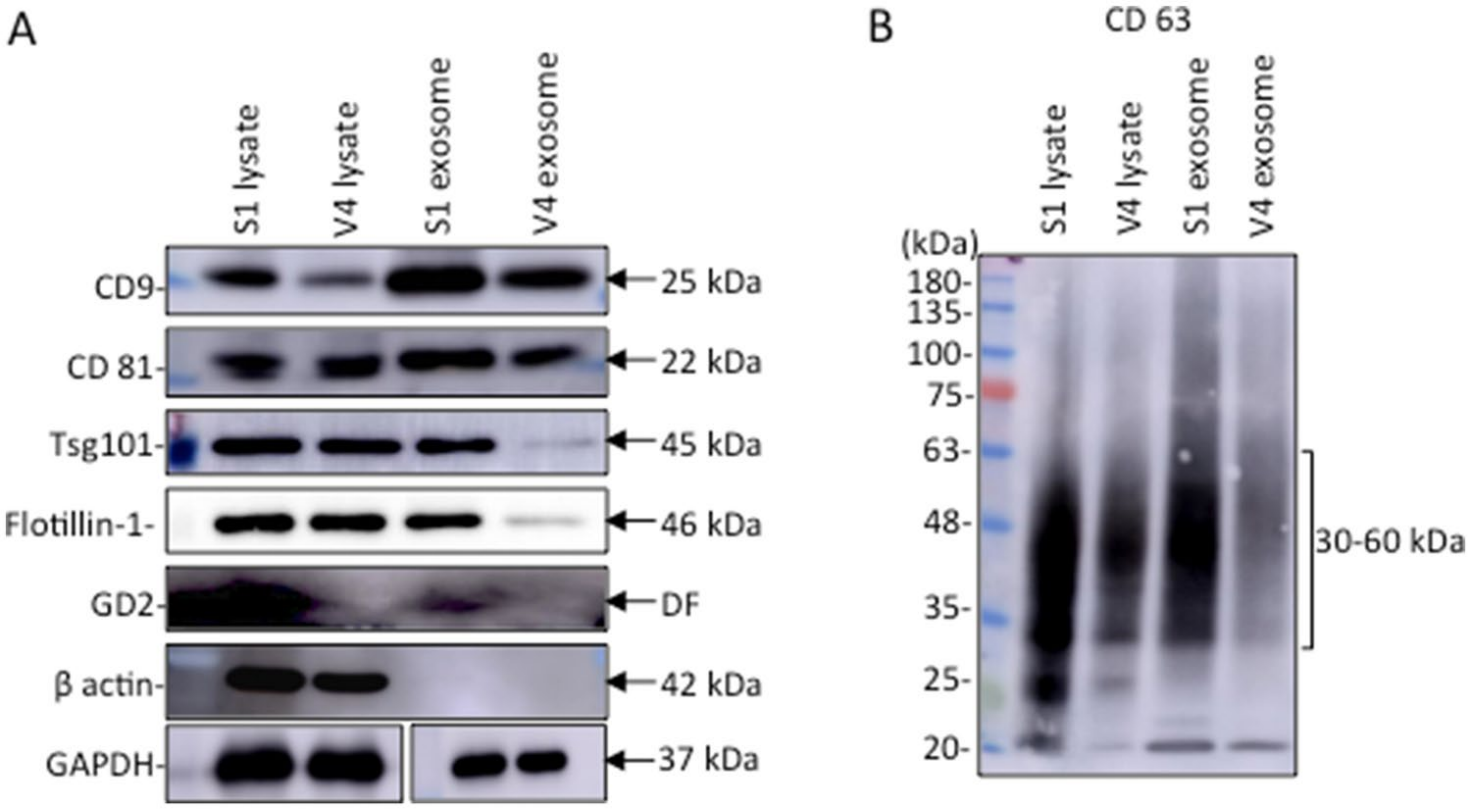
Immunoblotting of EV markers and a lipid raft marker. Immunoblotting of cell lysates and EVs from GD2+ S1 cells and GD2- V4 cells was performed using specific mAbs for the individual molecules. Anti-GD2 and anti-β-actin antibodies were also used (**A**). The results of immunoblotting with anti-CD63 are shown separately in (** B**). Lysates from GD2+ S1 and GD2- V4 cells and lysates from S1- and V4-derived exosomes were subjected to SDS‒PAGE. Subsequently, immunoblotting was performed using antibodies reactive with EV markers, e.g., CD9, CD81, Tsg101, CD63, a lipid raft marker Flotillin-1 (all used at 1:1000 dilution), GD2 (1:100 dilution of ascites), and β-actin (1:1000 dilution) and GAPDH (1:1000 dilution). An ImmunoStar LD detection kit was used for the detection of bands using Amersham Imager (Model: 680 software version 2.0, GE Healthcare, Uppsala, Sweden). These results are the representative data of repeated experiments with similar patterns.

EV markers and a lipid raft marker showed different levels in EVs from S1 and V4, while their levels showed mild differences in the lysates of the two cell lines. Immunoblotting of cell lysates and EVs from GD2+ S1 cells and GD2- V4 cells was performed using specific monoclonal antibodies (mAbs) for markers of EVs and lipid rafts. Anti-GD2, anti-β-actin and anti-GAPDH antibodies were also used. The results revealed that cell lysates from GD2+ S1 and GD2- V4 cells contained almost equivalent levels of CD81, Tsg101, and Flotillin-1 (a marker of lipid rafts) except for CD9 and CD63, as well as β-actin and GAPDH (Fig. 2A,B). However, lysates from S1-derived EVs showed high levels of these marker proteins compared with those from V4 cells. In particular, Tsg101, Flotillin-1 and CD63 showed big differences between S1 and V4.

**Figure 3:**
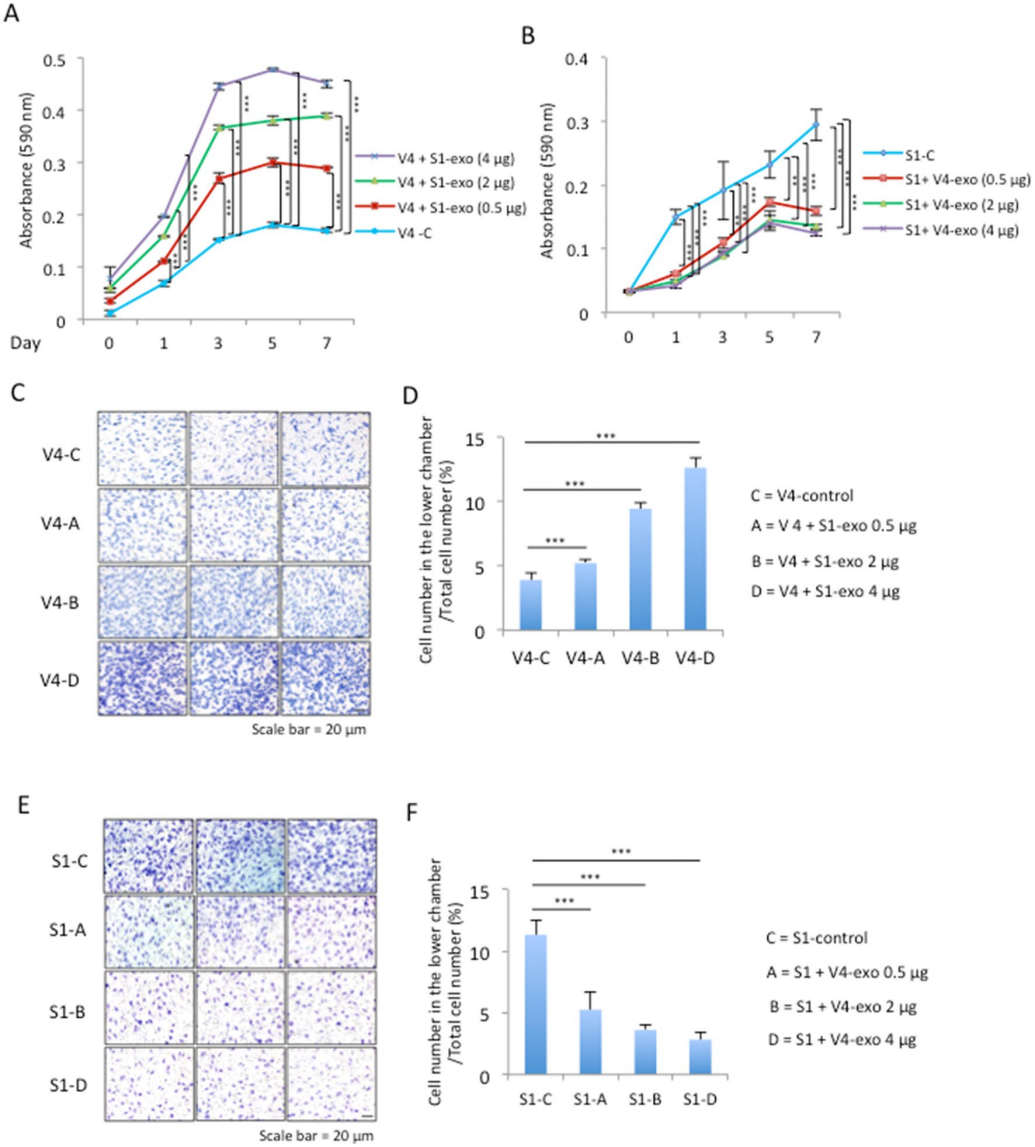
EVs derived from GD2+ S1 cells markedly enhanced the cell growth and invasion of GD2- V4 cells. (**A**, **B**) Proliferation was analyzed using GD2- V4 cells and GD2+ S1 cells by MTT assays. Cells (3 × 10^3^) were seeded in each well of 96-well plates. Then, EVs (0.5, 2.0 and 4.0 μg) derived from S1 cells were added to V4 cells (**A**), and EVs (0.5, 2.0 and 4.0 μg) derived from V4 cells were added to S1 cells (**B**). PBS alone was added to control cells. MTT solution (5 mg/ml in PBS) was added and incubated at 37 °C for 4 h on each day as indicated. After vigorous pipetting, the absorbance was measured at 590 nm, and the relative absorbance was plotted. The analysis was performed in triplicate, and the mean ± SD is presented. (**A**, **B**) The data at day 1, 3, 5 and 7 were analyzed by two-way ANOVA with a Tukey post hoc test. *P < 0.05, **P < 0.01, and ***P < 0.001. (**C**–**F**) Invasion was examined with cell culture inserts using GD2+ S1 and GD2- V4 cells. The upper chamber of the inserts was coated with 200 μg/ml Matrigel. After 24 h of incubation, noninvaded cells were removed, invaded cells in the lower chamber were stained with Giemsa, and the number of cells was counted under a microscope. (**C**, **E**) Microscopic images of invaded cells from the upper chambers where GD2- V4 and GD2+ S1 cells were plated, followed by the addition of S1-derived EVs and V4-derived EVs, respectively. (**D**, **F**) The analysis was performed in triplicate, and the mean ± SD is presented. The data were analyzed with an unpaired Student’s two-tailed t test. ***P < 0.001. Scale bar: 20 μm.

EVs from GD2-expressing cells enhanced the growth and invasion of GD3-negative cells. GD2-negative V4 cells showed increased cell growth when treated with GD2-expressing S1-derived EVs in a dose-dependent manner (Fig. 3A). However, when EVs released from GD2-negative cells (V4) were added to the culture medium of GD2-expressing cells (S1), growth of S1 cells was dose-dependently suppressed (Fig. 3B). When GD2- V4 cells were cultured with EVs released from GD2+ S1 cells, an invasion assay using Boyden chambers revealed that the invasion gradually increased along with the added dose of EVs, as shown in Fig. 3C and D. When GD2+ S1 cells were treated with GD2- V4-derived EVs, S1 cells showed reduced invasion in a dose-dependent manner (Fig. 3E,F).

**Figure 4:**
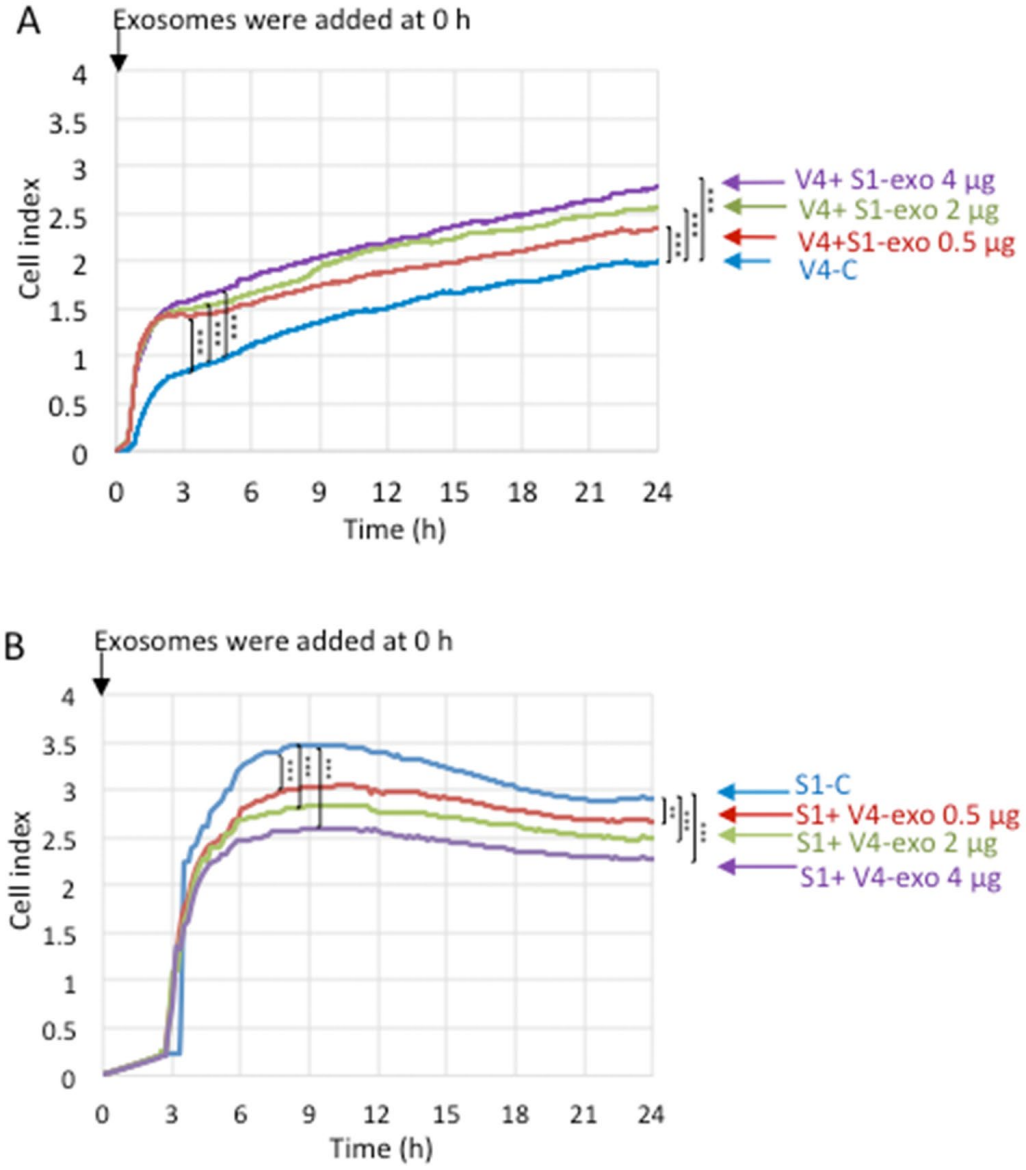
Effects of EVs on the adhesion of GD2- and GD2 + cells. (**A**, **B**) Cell adhesion was investigated using GD2- V4 cells and GD2+ S1 cells in collagen I-precoated (5 μg/mL in PBS, 100 μL/well) 16-well microplates by an RT-CES system. Cells (1 × 10^4^) were seeded in each well of the plates containing 100 μl of culture medium, EVs (0.5, 2.0 and 4.0 μg) derived from S1 cells were added to V4 cells at 0 h (**A**), and EVs (0.5, 2.0 and 4.0 μg) derived from V4 cells were added to S1 cells at 0 h (**B**). PBS was added to control cells. The data at 3 h and 24 h incubation (**A**) or at 7.5 h and 24 h (**B**) were analyzed with an unpaired Student’s two-tailed t test. **P < 0.01 and ***P < 0.001.

Cell adhesion of GD2- V4 cells was enhanced by the addition of EVs from GD2+ S1 cells. With a real-time cell electron sensing (RT-CES) system, the effects of EVs on cell adhesion to the extracellular matrix (ECM) were examined. When V4 cells were treated with EVs derived from S1 cells, cell adhesion, as presented by the cell index (CI), was increased depending on the amounts of added EVs (Fig. 4A). In contrast, when S1 cells were treated with EVs derived from V4 cells, cell adhesion was suppressed depending on the amounts of EVs used (Fig. 4B). In these experiments, EVs were added to wells together with cells at time 0.

**Figure 5:**
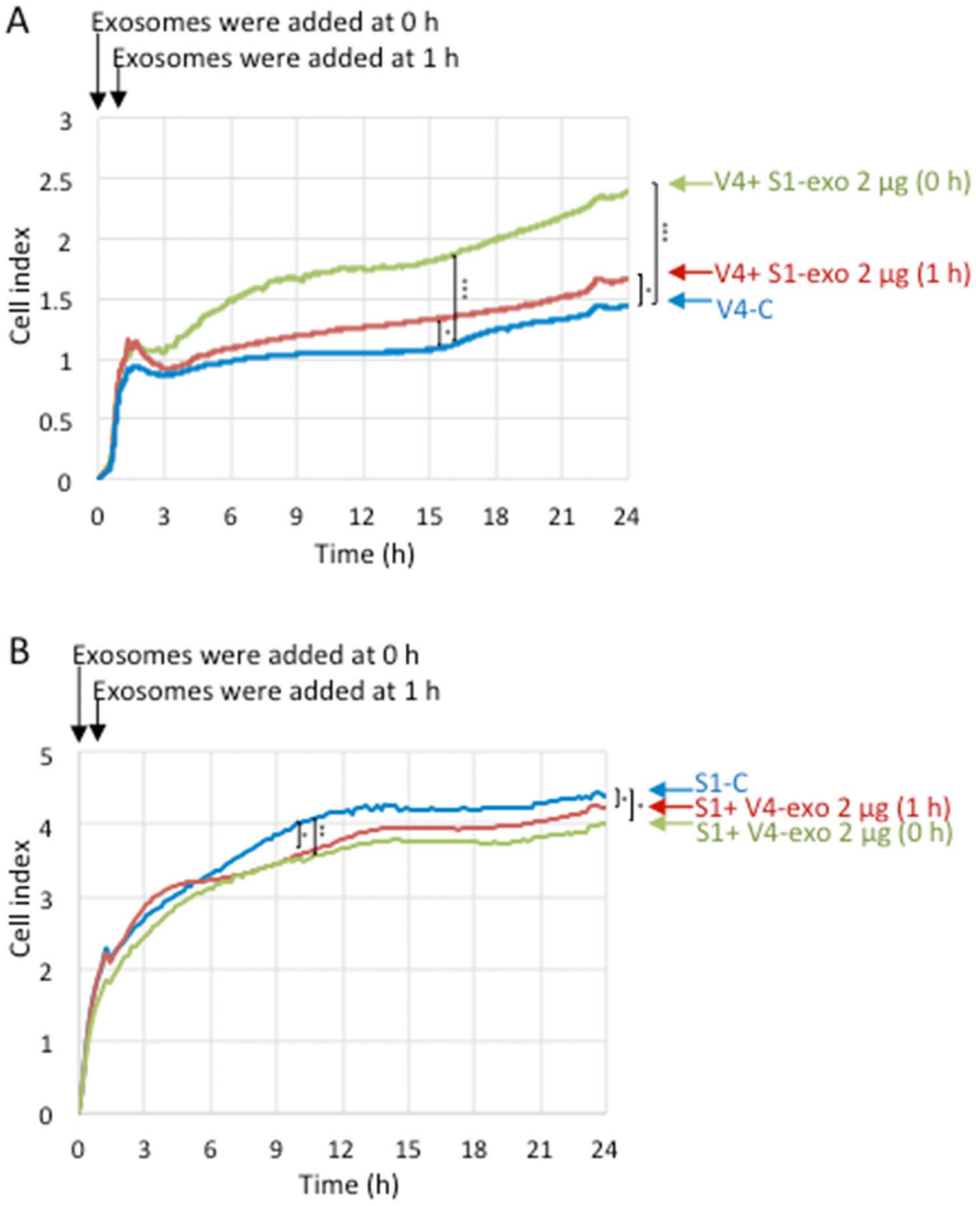
Effects of EVs on the adhesion of GD2- and GD2 + cells at 0 h and 1 h. (**A**, **B**) Examined effects of EVs added at 0 h and 1 h on cell adhesion using GD2- V4 cells and GD2+ S1 cells in collagen I-precoated (5 μg/mL in PBS, 100 μL/well) 16-well microplates by the RT-CES system. V4 or S1 cells (1 × 10^4^) were seeded in each well of the plates containing 100 μl of culture medium. Then, EVs (2.0 μg) derived from S1 cells were added to V4 cells at 0 h and 1 h (**A**), and EVs (2.0 μg) derived from V4 cells were added to S1 cells at 0 h and 1 h (**B**). PBS was added to control cells. Differences in cell adhesion (CI) between nontreated cells and EV-treated cells at 0 h and 1 h were analyzed, and the data at 15 h and 24 h incubation (**A**), and 10.5 h and 24 h (**B**) were analyzed with an unpaired Student’s two-tailed t test. *P* values are indicated as *P < 0.05; **P < 0.01 and ***P < 0.001.

The effects of EVs on cell adhesion were stronger when added at time 0 of cell plating than when added at 1 h after cell plating. To compare the impact of EVs between the two timings of addition to plates, we added EVs to RT-CES plates at time 0 or at 1 h after cell plating. As shown in Fig. 5, both modes of addition resulted in similar effects on the cell index, i.e., S1-derived EVs enhanced V4 adhesion (Fig. 5A), while V4-derived EVs suppressed S1 adhesion (Fig. 5B). However, the addition of S1 EVs at time 0 was more efficient than that 1 h later in the enhancement of V4 cell adhesion (Fig. 5A).

**Figure 6:**
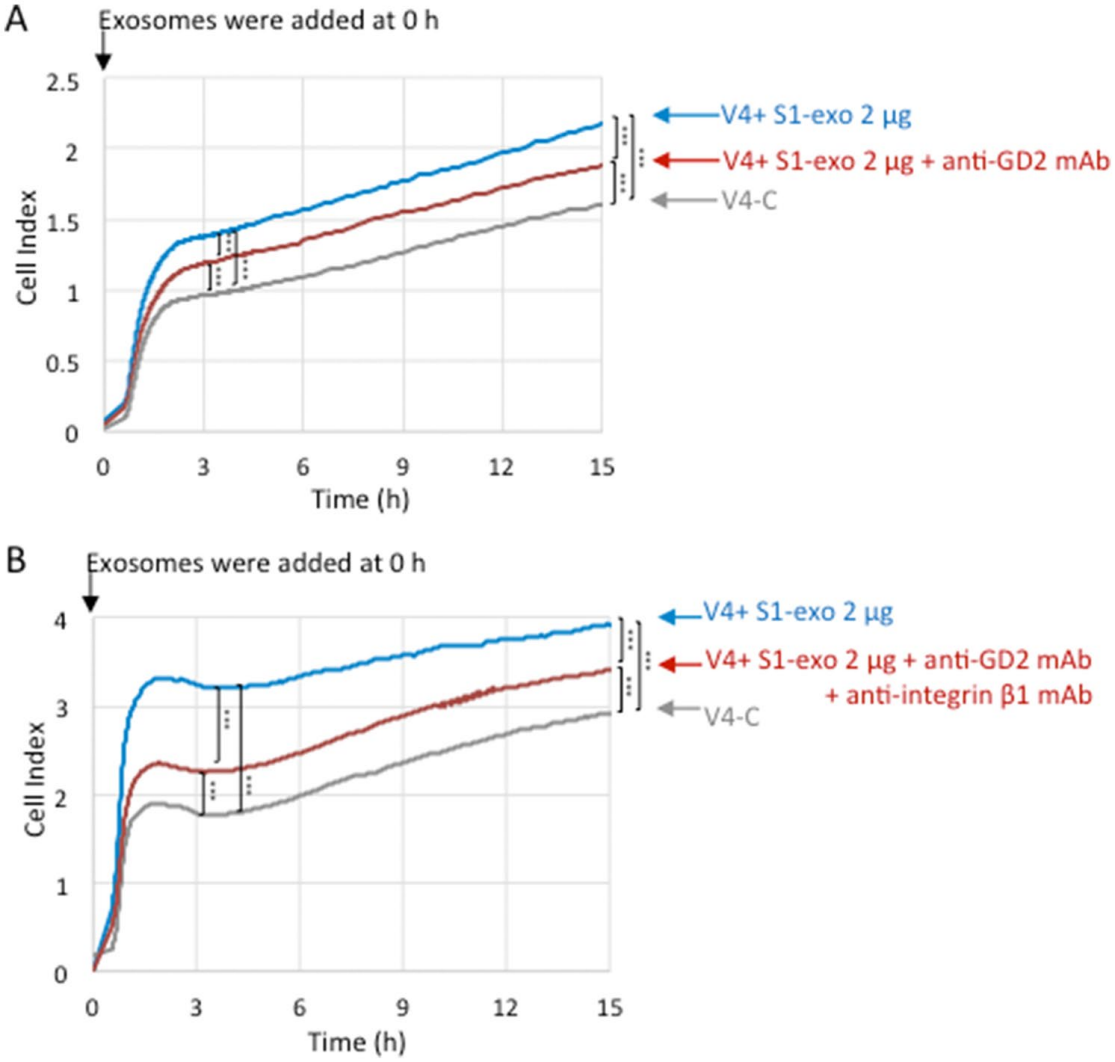
Effects of treatment with anti-GD2 mAb on the action of EVs during cell adhesion of GD2- V4 cells. (**A**) Effects of EVs with or without anti-GD2-mAb on cell adhesion were investigated by an RT-CES system using GD2- V4 cells in collagen I-precoated (5 μg/mL in PBS, 100 μL/well) 16-well microplates. V4 cells (1 × 10^4^) were seeded in each well of plates containing culture medium (100 μl), and exosomes (2.0 μg) derived from S1 cells were added to V4 cells at time 0. PBS was added to control cells. Then, anti-GD2 mAb (**A**) or anti-GD2 mAb and anti-integrin β1 mAb (**B**) were added to the wells as indicated in the figure. PBS was used as a control. Differences in cell adhesion were compared, and the data at 3 h and 15 h incubation (**A**, **B**) were analyzed with an unpaired Student’s two-tailed t test. *P* values are indicated as ***P < 0.001.

GD2 and integrins are involved in the action of EVs during cell adhesion. To investigate the involvement of GD2 on EVs, we added anti-GD2 mAb (220–51) to the S1-derived EVs at time 0 of RT-CES. Consequently, the GD2- V4 cells treated with S1-derived EVs showed a clearly increased cell index (CI) from the early stage until 15 h of observation compared with the nontreated V4 cells (Fig. 6). When a mixture of anti-GD2 mAb and S1-EVs was added to V4 cells, CI was persistently reduced (Fig. 6A), while it was still higher than that in the nontreated V4 cells. Furthermore, anti-integrin β1 mAb was added to EVs as well as anti-GD2 mAb to V4 cells, and CI was largely suppressed, particularly during the first few hours (Fig. 6B).

**Figure 7:**
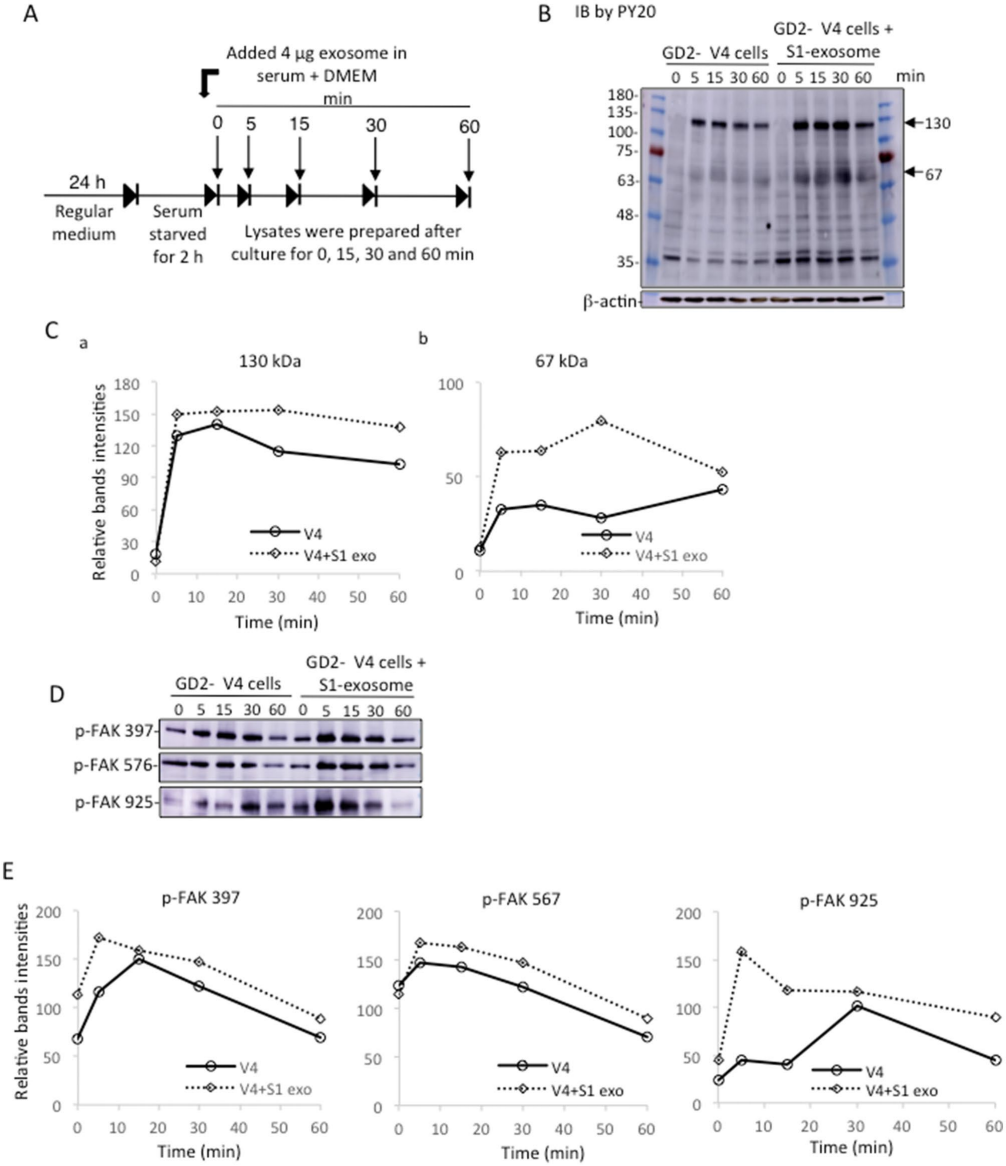
Tyrosine-phosphorylated protein levels in V4 cells increased after treatment with S1-EVs in DMEM during cell growth. (**A**) Diagram of preparing cells to obtain lysates during cell growth in DMEM containing FBS in the presence or absence of S1-EVs at various time points after plating. (**B**) After detaching with 0.02% EDTA/PBS, GD2- V4 cells (5 × 10^5^) were placed in 6 cm dishes with regular medium for 24 h at 37 °C. Cells were washed with plain DMEM, and starved in serum-free DMEM at 37 °C for 2 h. Then cells were cultured in the presence or absence of S1-derived EVs (4 µg) in serum + DMEM, and incubated for 0, 5, 15, 30 and 60 min at 37 °C. After incubation, the cells were lysed, and the lysates were used for SDS‒PAGE. Subsequently, immunoblotting was performed with the anti-phosphotyrosine mAb PY20. (**C**) Band intensities (130 and 67 kDa) in (**B**) were measured and plotted using ImageJ (1.53t). (**D**) Lysates as mentioned in (**B**) were subjected to SDS‒PAGE and immunoblotted with individual anti-FAK mAbs (p-397FAK, p-576FAK, p-925FAK mAbs). Band intensities were measured by ImageJ (1.53t) and plotted in (**E**).

Phosphotyrosine proteins were increased in GD2- V4 cells by the addition of GD2+ S1-derived EVs. To investigate the effects of EVs on serum stimulation-induced phosphorylation of intracellular proteins, we performed immunoblotting using cell lysates treated with serum-containing DMEM and EVs (Fig. 7). When V4 cells were cultured in FCS-containing medium, few weak bands were detected by the mAb PY20 from 5 min after stimulation (Fig. 7B left). Surprisingly, strong bands could be detected when V4 cells were treated with serum+ DMEM and S1-derived EVs at 5 ~ 30 min after serum stimulation (Fig. 7B right). The two major bands in Fig. 7B were scanned and are presented in Fig. 7Ca and Cb. Phosphorylated focal adhesion kinase (FAK) was also examined using 3 mAbs for tyrosine-phosphorylated FAK at different sites (Fig. 7D). The band intensities are plotted in Fig. 7E, showing stronger intensities in the S1-EV-treated samples in all situations.

**Figure 8:**
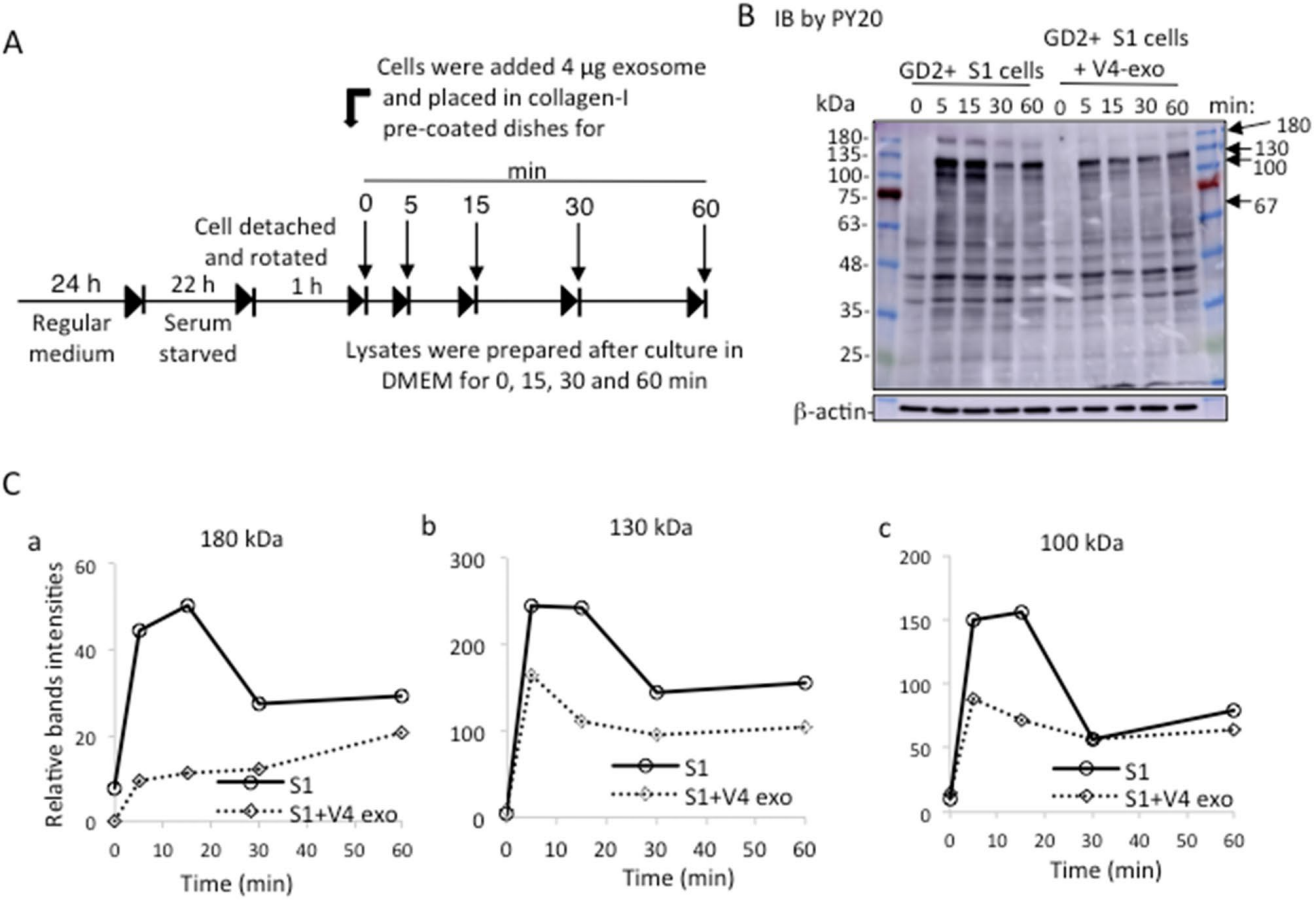
Tyrosine-phosphorylated protein levels were decreased after treatment of S1 cells with V4-EVs in DMEM during cell adhesion. (**A**) Diagram of preparing cells to obtain lysates during cell adhesion in DMEM in the presence or absence of V4-EVs for various time points. (**B**) GD2+ S1 cells were cultured in regular DMEM at 37 °C for 24 h. Cells were washed with plain DMEM and starved in serum-free DMEM at 37 °C for 22 h, and then cells were detached by 0.02% EDTA/PBS. Cell suspension was rotated at 37 °C for 1 h, and S1 cells were placed in collagen 1-pre-coated plate with DMEM in the presence or absence of V4-derived EVs (4 µg) and incubated for 0, 5, 15, 30 and 60 min at 37 °C. After incubation, the cells were lysed, and the lysates were used for SDS‒PAGE. Subsequently, immunoblotting was performed using the anti- phosphotyrosine mAb PY20. (**C**) Band intensities (180, 130, 100 kDa) in (**B**) were measured and plotted using ImageJ (1.53t).

Phosphotyrosine proteins were reduced in GD2+ S1 cells by the addition of GD2- V4-derived EVs. To investigate the effects of EVs on intracellular signaling during cell adhesion, we performed immunoblotting of cell lysates using the anti-p-tyrosine mAb PY20 (Fig. 8). After pretreatment of S1 cells, they were plated in collagen-precoated dishes with or without V4-derived EVs at time 0, and cell lysates were prepared as shown in Fig. 8A and used in immunoblotting with the mAb PY20. Phosphotyrosine bands were markedly reduced at 5 ~ 15 min compared with those of the nontreated S1 lysates (Fig. 8B). Major components of tyrosine-phosphorylated bands, e.g., 180 kDa, 130 kDa and 100 kDa, showed apparent decreases in the band intensities, as shown in Fig. 8C.

## Discussion

Although a number of biological functions of EVs have been reported in various experimental and clinical situations^31,42^, their roles differ depending on the primary features of the secretory cells and recipient cells. For example, EVs from mesenchymal stem cells, immune cells, or various cancer cells have been extensively studied, and intriguing findings have been reported to date^43^. In particular, EVs released from cancers have been shown to play important roles in the regulation of normal cells around cancer tissues. Recently, glycosylated molecules on EVs were analyzed by lectin array, showing characteristic expression patterns of lectin-recognizing carbohydrates^44^. For example, from mannose-binding lectins, glycans characteristic of CD61 were identified specifically in EVs of Alzheimer disease patients^45^. Many of those glycans detected by the lectin array approach seem to be present on glycoproteins. For glycosphingolipids in EVs, either expression or function have not been well analyzed thus far. Only one study has been published, showing effects of EVs released from human melanocytes transfected by GD3 synthase cDNA on normal melanocytes^46^. Consequently, enhancement of mobility was observed in melanocytes treated with EVs from GD3-expressing melanocytes.

Although it became possible to investigate phenotypes of glyco-remodeled cells and animals, the mechanisms by which gangliosides alter the cell features and signals have not been clearly demonstrated. The most important issue for this status is the fact that glycosphingolipids are expressed on the outer layer of the lipid bilayer membrane^4^. Therefore, it is difficult to explain how GSLs mediate cell signals that are introduced via the cell membrane. We have solved this issue by identifying interacting molecules with some target gangliosides on the cell surface by the EMARS/MS method^47^. To identify GD2-associated membrane molecules, we performed EMARS/MS, and integrin β1 was identified as a sole molecule detected only in GD2-positive melanoma cells^41^. We demonstrated that GD2 and integrins form molecular complexes in lipid rafts and cooperate to enhance the malignant properties of melanomas by increasing the phosphorylation of EGFR and FAK^41^. GD2 was reported to be expressed at advanced stages of melanomas, e.g., the metastatic stage^22^. Therefore, the results reported in this article seemed to correspond with the supposed roles of GD2 in the later phase of melanoma progression^48^.

In this study, we analyzed the effects of EVs derived from GD2-positive melanoma cells on the phenotypes of GD2-negative melanoma cells, showing rapid activation of the recipient cells via increased cell growth, invasion, and cell adhesion in a dose-dependent manner (Figs. 3 ~ 7). Furthermore, exosomes added to GD2-negative cells induced rapid phosphorylation of various signaling molecules, e.g., EGFR (180 kDa) and FAK (130 kDa) as we previously demonstrated using mass spectrometry^41^. These effects of exosomes on the target cells were quite similar to the effects of GD2 expression compared with GD2-negative cells. Cooperation of GD2 with integrins might affect EVs, as shown by us on a cellular basis^41^. Thus, many GD2 functions in melanomas might be carried through EVs. Interestingly, GD2-expressing melanoma cells underwent suppression of the malignant phenotypes when they were treated with GD2-negative (GM3 only) melanoma cell-derived EVs. These results suggested that GM3-positive melanoma cells weakly or reversely regulate surrounding cells via released EVs.

The results obtained in this study seem to propose many suggestions on the roles of GD2 in EVs in the induction of malignant properties of cancer cells and normal cells. As shown in Fig. 6, anti-GD2 mAb and anti-integrin Ab showed apparent suppression of the exosome effects on the enhancement of cell adhesion in RT-CES, suggesting that GD2 on the exosomes play important roles during the targeting of exosomes toward target cell membrane. Attachment of exosomes and subsequent triggering of intracellular signals should be exerted via GD2 on EVs probably as a cooperation with integrins. Furthermore, the presence of GD2 in EVs might be involved in the regulation of the process of EV generation in intraluminal vesicle and multivesicular body^49^, since GD2+ EVs contain much more Tsg101 and some tetraspanins that are considered to play roles in the generation of EVs.

The facts that cancer-derived exosomes brought about a high incidence of bone marrow metastasis when injected before melanoma cell injection^29^ or breast cancer-derived EVs with different integrin isoform expression played important roles in the niche formation for distant metastasis determining metastatic organ tropism^30^ suggest that GD2 and integrins also cooperate on EVs. In addition to the coexpression of GD2 and integrins on melanoma cells, as shown by flow cytometry, coexpression of these two molecules on EVs was analyzed by immune-EV chemistry by fixing EVs on glass-bottom plates. Interestingly, heterogeneity in the expression patterns of GD2 and integrin β1 was observed, i.e., double-stained vesicles and single-stained vesicles of either molecule (data not published). Heterogeneity in EVs has been reported by many researchers and still requires further investigation^50^. The interaction and cooperation of GD2 and integrins on EVs remain to be investigated in the future.

The clinical application of the results obtained in this study will be promising for the construction of novel strategies to overcome cancers. This is especially true because GD2 has drawn increasing attention from both basic researchers and clinicians because of its unique function, such as a cancer-stem marker^51^, and because it is an efficient target of immune therapy^52^.

## Methods

### Cell lines and cell culture

The human melanoma cell SK-MEL-28 subline N1^53^ (provided by K.O. Lloyd at Memorial Sloan-Kettering Cancer Center, New York) was used to establish a ganglioside GD2-expressing S1 clone by transfecting cDNAs of *ST8SIA1*^54^ and *B4GALNT1*^55^. The V4 clone was established as a vector control^48^ (Fig. 1A). The S1 clone was grown in Dulbecco’s minimal essential medium (DMEM) containing 7.5% fetal bovine serum (FBS or FCS) and G418 (600 μg/ml), and the vector control V4 clone was grown in DMEM containing 7.5% FBS and G418 (400 μg/ml) at 37 °C in a humidified atmosphere containing 5% CO_2._

### Antibodies and reagents

Anti-GD2 mAb, 220–51 was generated in our laboratory^56^. The other antibodies and reagents were obtained from commercial sources as follows: fluorescein isothiocyanate (FITC)-conjugated goat anti-mouse IgG (H + L) (Cat. No. 55514) from Cappel (Durham, NC, USA) and horseradish peroxidase (HRP)-conjugated anti-mouse IgG antibody (Cat. No. 7076S), HRP-conjugated anti-rabbit IgG antibody (Cat. No. 7074S), GAPDH rabbit mAb (Cat. No. 2118) from Cell Signaling Technology (Danvers, MA, USA). Mouse anti-integrin β1 mAb (Cat. No. sc-374429) and anti-Flotillin-1 mAb (Cat. No. sc-74566) were purchased from Santa Cruz Biotechnology (Dallas, TX, USA). Mouse anti-phosphotyrosine antibody PY20 (Cat. No. sc-508) was purchased from Santa Cruz Biotechnology. Monoclonal anti-β actin antibody (A5441-2ML) was purchased from Sigma-Aldrich (St. Louis, MO, USA). Anti-phospho-FAK (Tyr-576)(Cat. No. sc-16563-R), Tyr-397(sc-81493), and Tyr-925) (sc-11766-R) and mouse Tsg101 (C-2) (Cat. No. sc-7964) antibodies were purchased from Santa Cruz Biotechnology. Anti-CD81 mAb (Cat. No. 66866-1-1 g) was purchased from Proteintech (Rosemont, IL, USA). Anti-CD9 (Cat. No. 014-27763) and anti-CD63 mAbs (Cat. No. 012-27063) were from Fujifilm Wako (Osaka, Japan). Giemsa (Cat. No. 079-04391) and BSA (Cat. No. LEH3163) were obtained from Wako (Osaka, Japan). Tim4-beads (Cat. No. 297-79701), PS Capture Exosome Flow Cytometry Kit) (Cat. No. CC050), and 2-Amino-2 hydroxymethyl-1, 3-propanediol (Cat. No. 201-06273) were from Fujifilm (Osaka, Japan), Matrigel (Cat. No. 354234) was from BD Bioscience (San Jose, CA, USA), and collagen-1 (Cat. No. CC050) and Tween20 (Cat. No. P2287) were from Sigma-Aldrich. Protease Inhibitor Mixture (Cat. No. 539131) was from Calbiochem (San Diego, CA, USA), and cell lysis buffer (Cat. No. 9803S) was from Cell Signaling Technology. The ImmunoStar LD (Cat. No. 290–69,904) detection kit, G418 (Cat. No. 076-05962), sodium hydrogen carbonate (Cat. No. 191–01,305), sodium dodecyl sulfate (Cat. No. 196–08,675), ammonium peroxodisulfate (Cat. No. 016-20501),* N*,* N*,* N*^1^ ,* N*^1^—tetramethyl-ethylenediamine (Cat. No. 205-06313) were from Wako (Osaka, Japan). MTT (3-(4,5-dimethylthiazol-2-yl) -2,5-diphenyltetrazolium bromide) (Cat. No. 349-01824) was from Dojindo laboratories, Kumamoto, Japan. Fetal Bovine Serum, REF 04-007-1A from B1 biological industries (Kibbutz Beit-Haemek, Israel), Gibco penicillin streptomycin (Cat. No. 15140-122) was from Thermofisher Scientific, USA. FastGene BlueStar prestained protein marker (Cat. No. MWP03-8) from Nippon Genetics Europe GmbH, Duren, Germany. Phenylmethylsulfonylfluoride (PMSF) (Cat. No. 10837091001) was from Roche Diagnostics GmbH, Mannheim, Germany.

### Preparation of exosome-free FCS isolation

First, we sterilized the Beckman polypropylene centrifuge tubes (REE-326823, Beckman Coulter, Brea, CA, USA) with 70% alcohol and dried in clean bench. FCS were poured into tubes up to 38.5 ml, and centrifuged at 4 °C at 175,000× g for 16 h by using Beckman SW32Ti rotor (Kent, MI, USA). The supernatants were collected in 50 ml falcon tubes and stored until use.

### Isolation of EVs from culture supernatants of melanoma cells

Near confluent cells (70–75%) in 15-cm dishes were washed three times with cold PBS and cultured with exosome-free FCS in DMEM (7.5% FCS in DMEM) for 48 h. Culture supernatants were collected in 50 ml tubes and centrifuged at 4 °C at 500 × g for 10 min. Supernatants were again centrifuged at 4 °C at 20,000 × g for 20 min. Supernatants were filtered using 0.22 μm Sartolab filters (RF-150) (Zartorius, Helsinki, Finland) and poured into Beckman polypropylene ultracentrifuge tubes (Beckman Coulter, Brea, CA, USA). Then, the samples were rotated at 175,000 g at 4 °C for 84 min, and the supernatants were discarded. Then, cold PBS was added, and the samples were rotated at 175,000 g at 4 °C for 84 min. After removal of the supernatants, the samples were slightly vortexed, and 200 μl of cold PBS was added. The samples were then used immediately or stored at -80 °C after aliquoting. Exosome sample in PBS was used for measuring proteins using Pierce BCA Protein Assay Kit (Cat. No. 23225, Thermofisher Scientific, Massachusetts, USA. DC protein assay kit (Bio-Rad, Hercules, CA, USA) was also used. To confirm that the preparations contain mainly exosomes, we performed size evaluation with NanoSight, and also immunoblotting of tetraspanins. A part of the results of size distribution with NanoSight has been attached as Supplementary Figure Fig. S1.

### Cell growth assay (MTT assay)

GD2+ S1 and/or GD2- V4 cells (3 × 10^3^) were placed in each well of 96-well plates with 100 μl of DMEM with 7.5% FBS. EVs (0.5, 2.0 and 4.0 μg) derived from V4 cells were added to S1 cells, and Evs (0.5, 2.0 and 4.0 μg) derived from S1 cells were added to V4 cells. PBS alone was added to control cells. During culture, 10 μl of MTT (3-(4,5-dimethylthiazol-2-yl)-2,5-diphenyltetrazolium bromide) (5 mg/ml in PBS) solution was added to each well of 96-well plates at Days 0, 1, 3, 5 and 7 and incubated for 4 h at 37 °C. The reaction was stopped by adding 110 μl of 1-propanol containing 0.1% NP-40 and 0.4% HCl. After vigorous pipetting, the absorbance was measured at 590 nm by an automatic microplate reader (Thermo Fisher Scientific, Type: 357, Shanghai, China).

### Invasion assay with the Boyden chamber method

Cell culture inserts (Transparent PET membrane, 24-well format, 8.0 μm pore size (Cat. No. 353097), Life Sciences, Durham, USA) were used to perform the invasion assay as described^40^. Matrigel (a solubilized basement membrane preparation extracted from EHS mouse sarcoma, BD Bioscience, 20 μl) in cold PBS (200 μg/ml) was applied to the upper chamber of the cell culture inserts and incubated for 2 h at room temperature for polymerization. After removal of PBS, the upper chamber was filled with 200 μl of serum-free DMEM and incubated for 1 h, and the lower chamber was filled with DMEM containing 10% FBS. After removal of the medium from the upper chamber, S1 or V4 cells (3 × 10^4^) in 200 μl of serum-free DMEM were added. Then, exosomes (0.5, 2.0 and 4.0 μg) derived from V4 cells were added to S1 cells, and exosomes (0.5, 2.0 and 4.0 μg) derived from S1 cells were added to V4 cells. Control cells were treated with no exosomes. Cells were incubated for 24 h at 37 °C in a humidified atmosphere containing 5% CO_2_. After incubation, invaded cells on the surface of the lower chamber were stained with Giemsa (Wako, Osaka, Japan), and the number of cells was counted under a microscope (IX73P1F, Olympus, Tokyo, Japan).

### Cell adhesion assay with real-time cell electron sensing (RT-CES)

A real-time cell electronic sensing system (RT-CES) (Wako Pure Chemical, Osaka, Japan) was used to analyze the cell adhesion assay as described^48^. Microelectronic cell sensor arrays were integrated into the bottom of the microplates (E-Plate (16X) code 583-84881, model number DC16-B-NT) (ACEA Biosciences, Inc., San Diego, CA, USA). The sensor provides information that increases electrical resistance (cell index) to indicate an increase in cell adhesion. E-Plates were coated with collagen-1 (CL-1) (5 μg/m in PBS, 100 μL/well) at room temperature (RT) for 1 h and blocked with 1% BSA/10% FBS in D-MEM (100 μL/well) at RT for 1 h. After blocking the wells, S1 or V4 cells (1 × 10^4^) were seeded in each well of 16-well plates containing culture medium. Then, exosomes (0.5, 2.0 and 4.0 μg) derived from V4 cells were added to S1 cells, and exosomes (0.5, 2.0 and 4.0 μg) derived from S1 cells were added to V4 cells at 0 h or 1 h. Control cells were not treated with exosomes. Simultaneously, a cell adhesion assay was performed by adding exosomes from S1 cells and anti-GD2 mAb or adding exosomes from S1 cells, anti-GD2 mAb and anti-integrin β1 mAb to V4 cells at 0 h. Changes in cell adhesion were monitored continuously and are expressed as the cell index (CI).

### Flow cytometry

Ganglioside GD2 expression levels on the cell membrane were analyzed as previously described^57–59^. Briefly, cells (5 × 10^5^) were trypsinized and washed twice with cold PBS and incubated with diluted primary antibody in PBS for 60 min on ice. After being washed twice with PBS, the cells were stained with FITC-conjugated goat anti-mouse IgG (H + L) (Cappel, Durham, NC) as a secondary antibody for 45 min on ice. Then, the cells were washed three times with PBS, and the relative expression levels were analyzed by an Accuri C6 Flow Cytometer (Accuri Cytometers, Inc., Ann Arbor, MI, USA). A control sample was prepared using nonrelevant mAbs with the same subclasses of individual primary antibodies. The CFlow Plus program was used for the quantification of positive cells.

### Cell lysate preparation

Cell lysates were prepared as previously described^41,59^. Cells were washed three times with PBS, then lysed with a cell lysis buffer (20 mM Tris–HCl, 150 mM NaCl, 1 mM Na2EDTA, 1% Triton X-100, 1 mM EGTA, 2.5 mM sodium pyrophosphate, 1 mM β-glycerophosphate, 1 mM Na_3_VO_4_, 1 µg/mL leupeptin) (Cell Signaling) with 1 mM phenylmethylsulfonyl fluoride (PMSF) and Protease Inhibitor Mixture (Calbiochem). Cell lysates were centrifuged at 12,500 rpm (Kubota 3740, Tokyo, Japan) for 10 min at 4 °C to remove insoluble cell debris, and proteins in the supernatants were measured using Pierce BCA Protein Assay Kit (Cat. No. 23225, Thermofisher Scientific, Massachusetts, USA.

### Western immunoblotting

After preparation of the cell lysates, proteins in lysates were separated by SDS‒PAGE using 10% agarose gels as previously described^41,59^. A sample buffer consisting of 100 mM Tris–HCl (pH 6.8), 1% SDS, 5% glycerol, 0.3% bromophenol blue, and 2.5% 2-mercaptoethanol was used. Separated proteins were transferred onto an Immobilon-P membrane (EMD Millipore, MA, USA), and blots were blocked for 1 h with 3% skim milk or bovine serum albumin (BSA) in PBS including 0.05% Tween-20. Then, the membrane was incubated with primary antibodies followed by HRP-labeled secondary antibodies for 45 min. Bands of proteins were visualized using ImmunoStar LD detection kits (Wako Osaka, Japan), and quantification of signal intensity was performed by ImageJ as explained below after taking images with Amersham Imager (Model: 680 software version 2.0, GE Healthcare, Uppsala, Sweden). The intensities of protein bands were measured using ImageJ software (1.53t) by subtraction of corresponding background of individual bands within the images. Then, resulting intensities of bands were plotted with corresponding time course (0, 5, 15, 30 and 60 min). We have confirmed that there are linear relationships between the amount of proteins measured and the immunoblotting signals within limited ranges of applied protein amounts in a number of past experiments.

### Statistical analysis

Statistical analyses were performed as previously described^48^. Data are presented as the means ± SDs. The data were analyzed by two-way ANOVA with a Tukey post hoc test or an unpaired Student’s two-tailed t test to compare mean values as indicated in the individual figure legends. *p* values of < 0.05 were considered significant. Statistical significance was analyzed using R software (version 3.6.3).

## Supplementary Information

Supplementary Information 1.

Supplementary Information 2.

Supplementary Information 3.

Supplementary Information 4.

Supplementary Information 5.

Supplementary Information 6.

## Data Availability

All data are available. Koichi Furukawa should be contacted if someone wants to request the data from this study.
