# Supplementary material for: Extracellular vesicles released from ganglioside GD2-expressing melanoma cells enhance the malignant properties of GD2-negative melanomas
Source: Sci Rep. 2023 Mar 27;13:4987. doi: 10.1038/s41598-023-31216-4 (PMC10042834; doi:10.1038/s41598-023-31216-4)
Supplement: Supplementary file 1 — Supplementary Information 1. [file 41598_2023_31216_MOESM1_ESM.docx]

Supplementary Figure SFig. 1.

NanoSight:


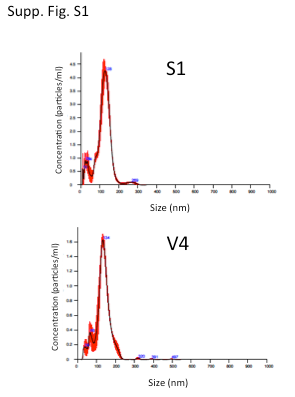
Nanoparticle Tracking Analysis (NTA)
Methods:
For detecting exosome content and size distribution, NTA was performed using the NanoSight NS3000 (Malvern, Worcestershire, UK). In brief, exosome samples were diluted with sterile PBS at 1:50~1:150, and an appropriate concentration was selected. The exosomes were injected at a constant flow rate at r.t, and three videos were acquired for each sample. The NanoSight NTA 3.2 software was employed for sample analysis.

Results: Exosomes derived from GD2+ cells (S1) and GD2- cells (V4) showed similar patterns with a single main peak. Mean and mode for V4 are 133.3 nm and 133.1 nm, and Mean and mode for S1 are 119.4 nm and 127.1 nm, respectively. Size distributions were also similar each other with a range between 80~180 nm.
