# Supplementary material for: Extracellular vesicles released from ganglioside GD2-expressing melanoma cells enhance the malignant properties of GD2-negative melanomas
Source: Sci Rep. 2023 Mar 27;13:4987. doi: 10.1038/s41598-023-31216-4 (PMC10042834; doi:10.1038/s41598-023-31216-4)

Fig. 2A: CD9 (L1-S1 lysate; L2-V4 lysate; L3-S1 exosome; and L4-V4 exosome).

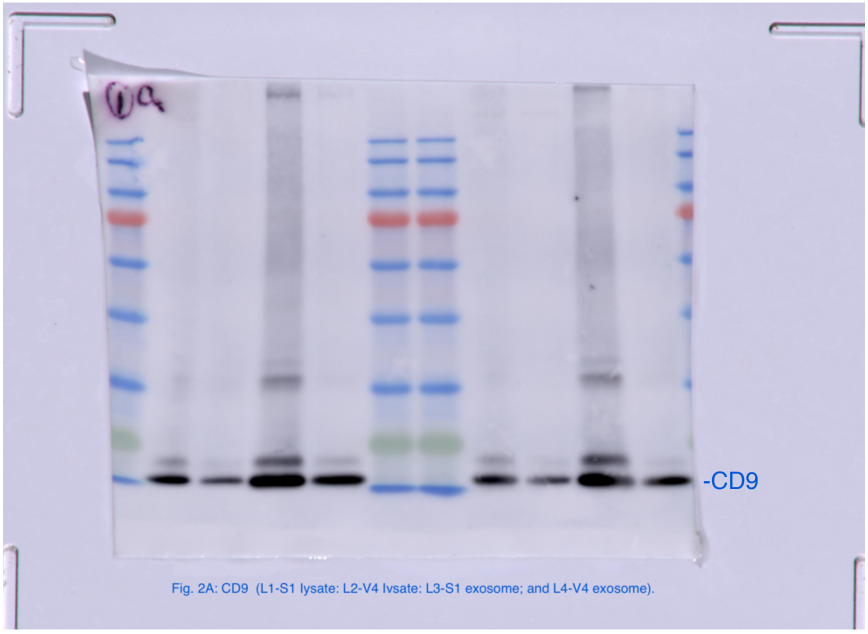

Fig. 2A: CD81 (L1-S1 lysate; L2-V4 lysate; L3-S1 exosome; and L4-V4 exosome).

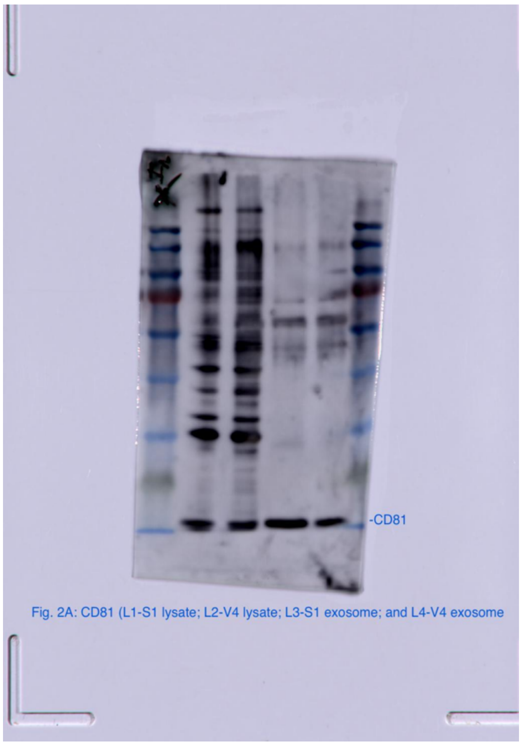

Fig. 2A: Tsg101 (L1-S1 lysate; L2-V4 lysate; L3-S1 exosome; and L4-V4 exosome).

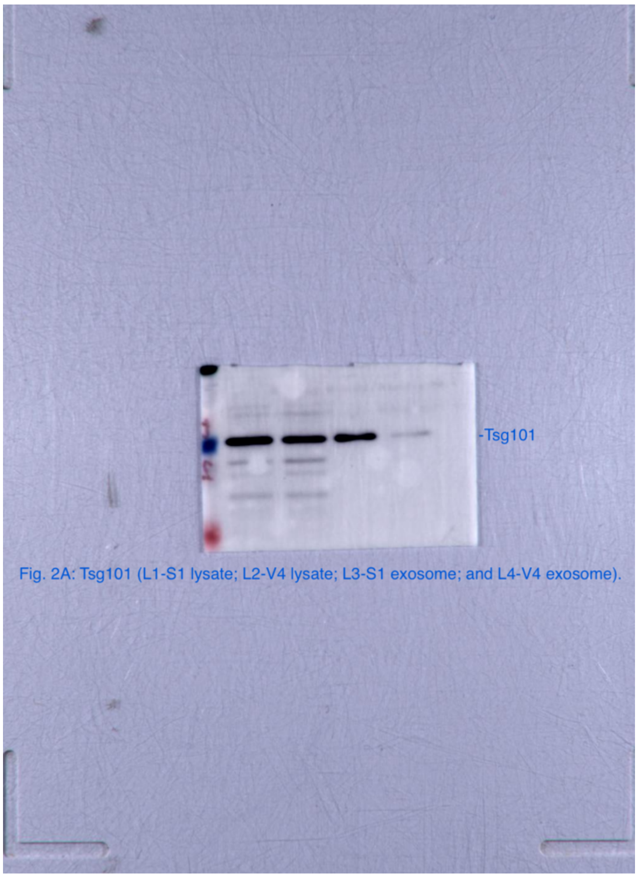

Fig. 2A: Flotillin-1 (L1-S1 lysate; L2-V4 lysate; L3-S1 exosome; and L4-V4 exosome).
(Image without colorimetric marker).

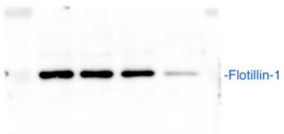

Fig. 2A: Flotillin-1 (L1-S1 lysate; L2-V4 lysate; L3-S1 exosome; and L4-V4 exosome).
(Image without colorimetric marker)

Fig. 2A: GD2 (L1-S1 lysate; L2-V4 lysate; L3-S1 exosome; and L4-V4 exosome).

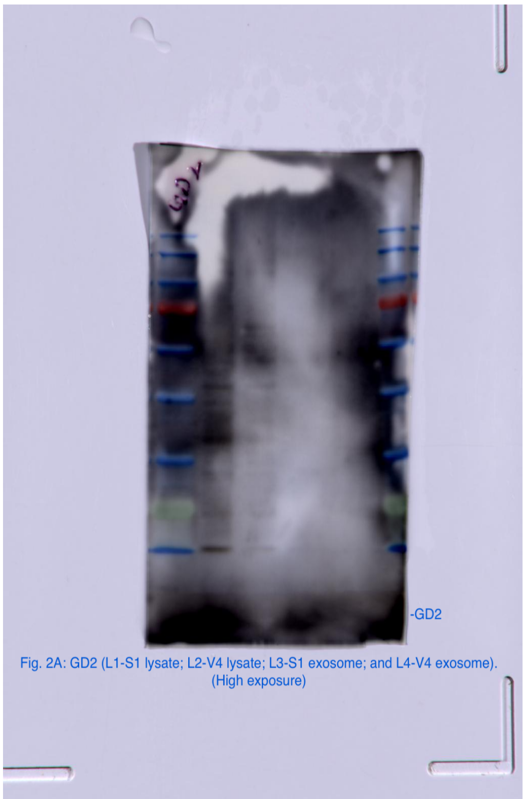

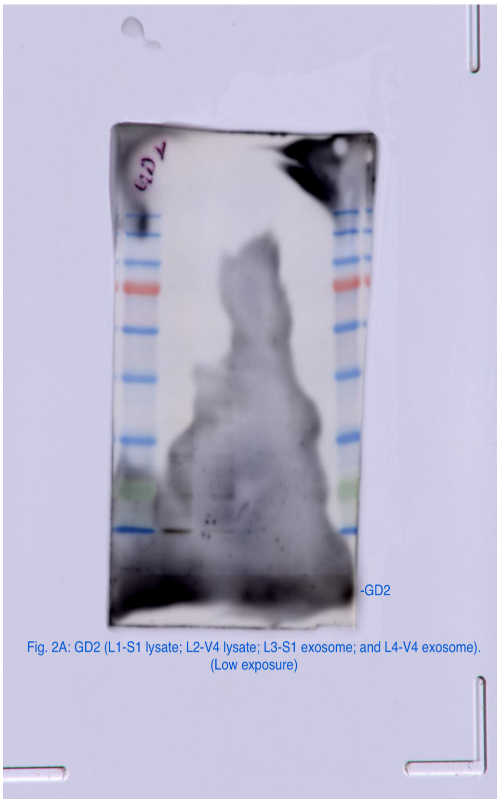

Fig. 2A: β-actin (L1-S1 lysate; L2-V4 lysate; L3-S1 exosome; and L4-V4 exosome).

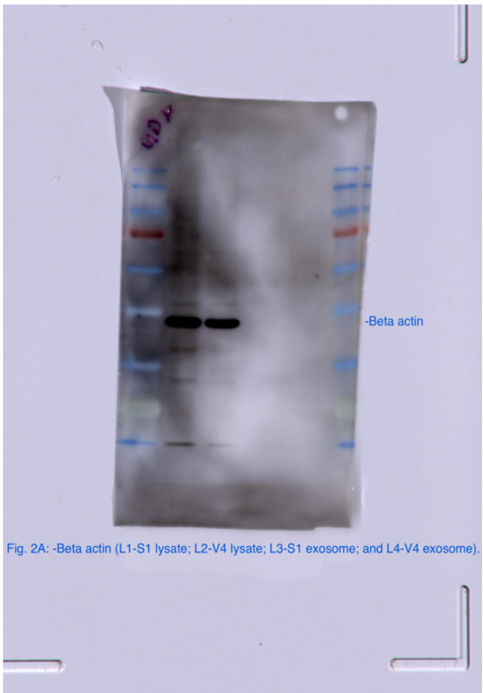

Fig. 2B: CD63 (L1-S1 lysate; L2-V4 lysate; L3-S1 exosome; and L4-V4 exosome).

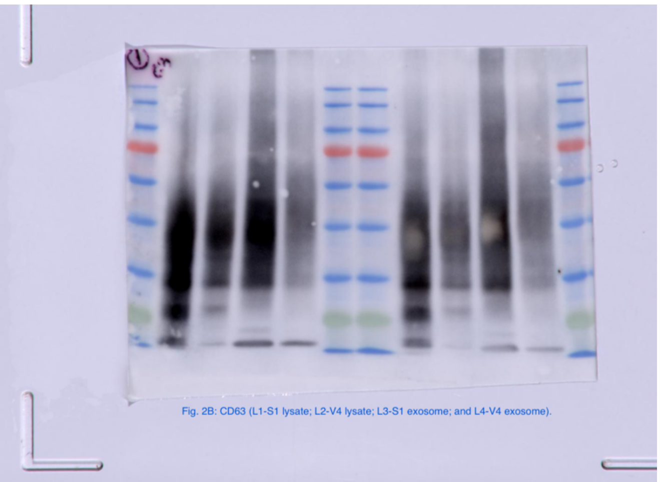

Supplement: Supplementary file 2 — Supplementary Information 2. [file 41598_2023_31216_MOESM2_ESM.pdf]
