# Supplementary material for: Extracellular vesicles released from ganglioside GD2-expressing melanoma cells enhance the malignant properties of GD2-negative melanomas
Source: Sci Rep. 2023 Mar 27;13:4987. doi: 10.1038/s41598-023-31216-4 (PMC10042834; doi:10.1038/s41598-023-31216-4)
Supplement: Supplementary file 3 — Supplementary Information 3. [file 41598_2023_31216_MOESM3_ESM.pdf]

Flotillin-1
1:2000

20220922KF Flotillin1 2022.09.27_12.45.00_Ch

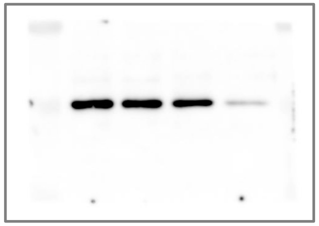

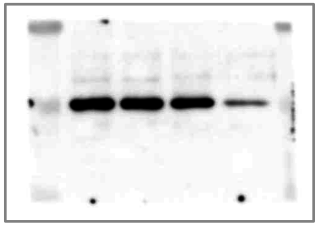

Exposed more to make the marker visible

Imager was used as black/white mode JPG

20220922KF Flotillin1 2022.09.27_12.45.00_Ch

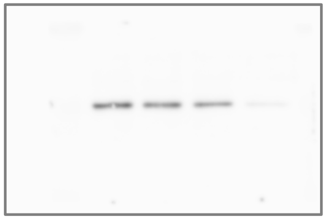

TIF

20221128
Image of whole Membrane
