# Supplementary figures and images for: Extracellular vesicles released from ganglioside GD2-expressing melanoma cells enhance the malignant properties of GD2-negative melanomas
Source: Sci Rep. 2023 Mar 27;13:4987. doi: 10.1038/s41598-023-31216-4 (PMC10042834; doi:10.1038/s41598-023-31216-4)

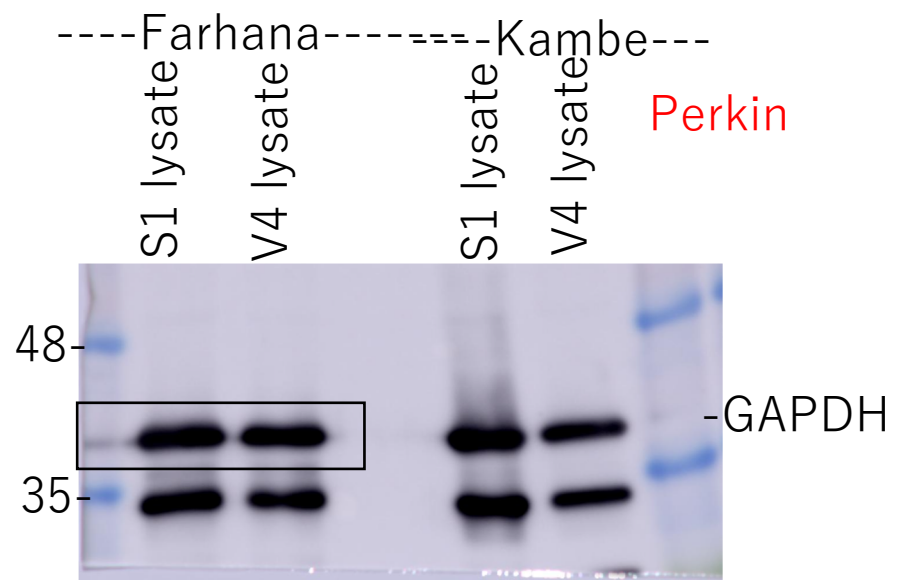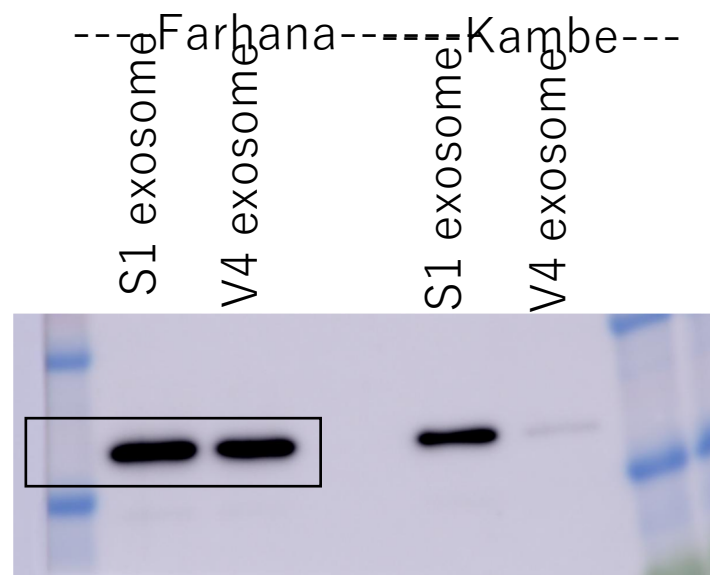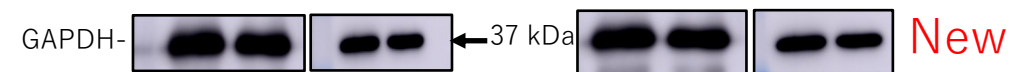

Supplement: Supplementary file 4 — Supplementary Information 4. [file 41598_2023_31216_MOESM4_ESM.pdf]
