# Supplementary material for: Extracellular vesicles released from ganglioside GD2-expressing melanoma cells enhance the malignant properties of GD2-negative melanomas
Source: Sci Rep. 2023 Mar 27;13:4987. doi: 10.1038/s41598-023-31216-4 (PMC10042834; doi:10.1038/s41598-023-31216-4)

**Fig. 7B:** Immunoblotting by PY 20 (Left- GD2-V4 cells: 0~60 min, and right- GD2-V4 cells + S1 exosome: 0~60 min).

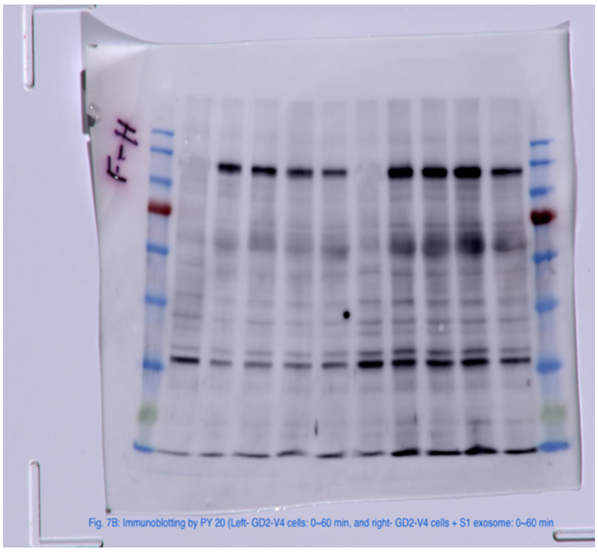

**Fig. 7B:** Beta actin (Left- GD2-V4 cells: 0~60 min, and right- GD2-V4 cells + S1 exosome: 0~60 min).

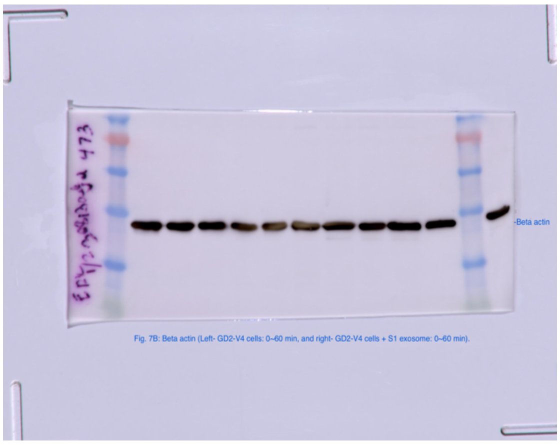

**Fig. 7D:** Immunoblotting with **anti-p-FAK 397 mAb** (Left- GD2-V4 cells: 0~60 min, and right- GD2-V4 cells + S1 exosome: 0~60 min).

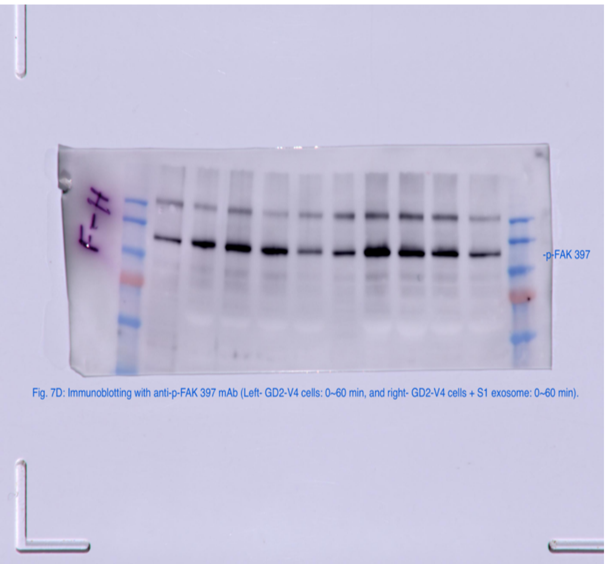

Fig. 7D: Immunoblotting with anti-p-FAK 397 mAb (Left- GD2-V4 cells: 0~60 min, and right- GD2-V4 cells + S1 exosome: 0~60 min).

**Fig. 7D:** Immunoblotting with **anti-p-FAK 576 mAb** (Left- GD2-V4 cells: 0~60 min, and right- GD2-V4 cells + S1 exosome: 0~60 min).

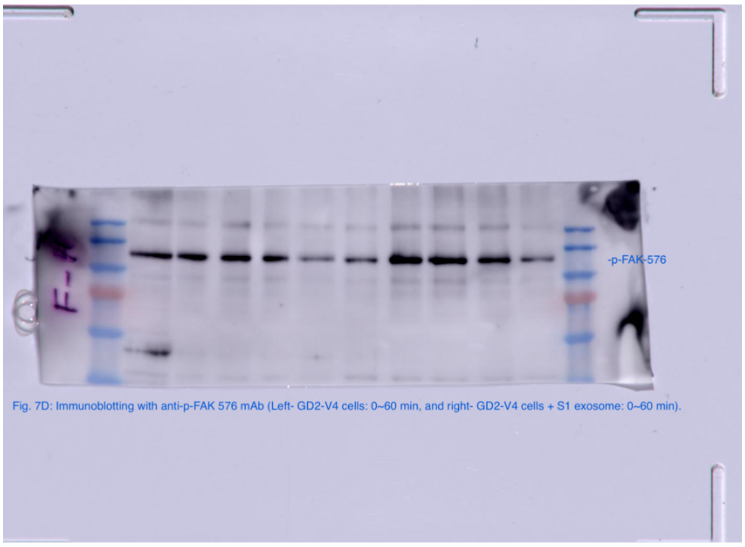

**Fig. 7D:** Immunoblotting with **anti-p-FAK 925mAb** (Left- GD2-V4 cells: 0~60 min, and right-GD2-V4 cells + S1 exosome: 0~60 min).

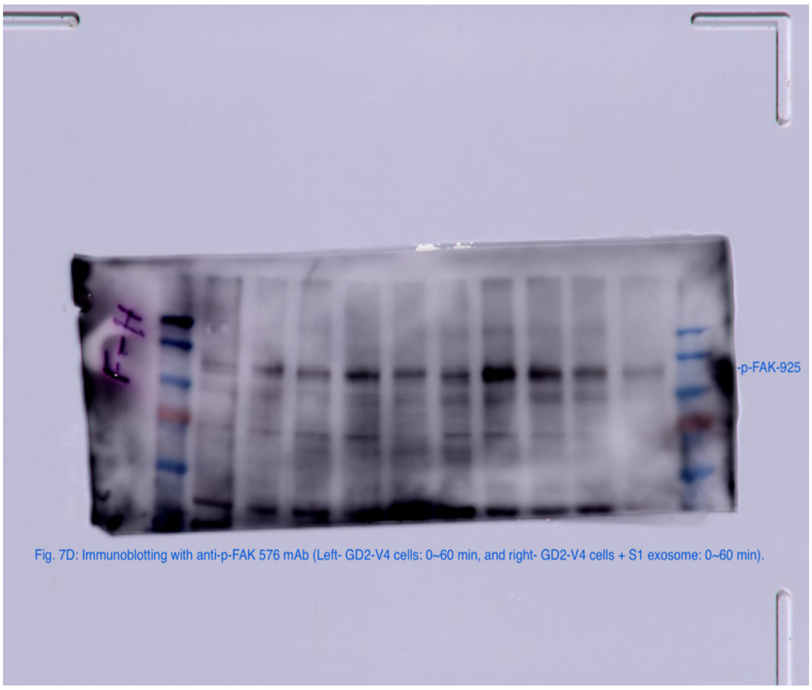

Supplement: Supplementary file 5 — Supplementary Information 5. [file 41598_2023_31216_MOESM5_ESM.pdf]
