# Supplementary material for: Extracellular vesicles released from ganglioside GD2-expressing melanoma cells enhance the malignant properties of GD2-negative melanomas
Source: Sci Rep. 2023 Mar 27;13:4987. doi: 10.1038/s41598-023-31216-4 (PMC10042834; doi:10.1038/s41598-023-31216-4)

Fig. 8B: Immunoblotting by PY 20 (Left- GD2+S1 cells: 0~60 min, and right- GD2+S1 cells + V4 exosome: 0~60 min).

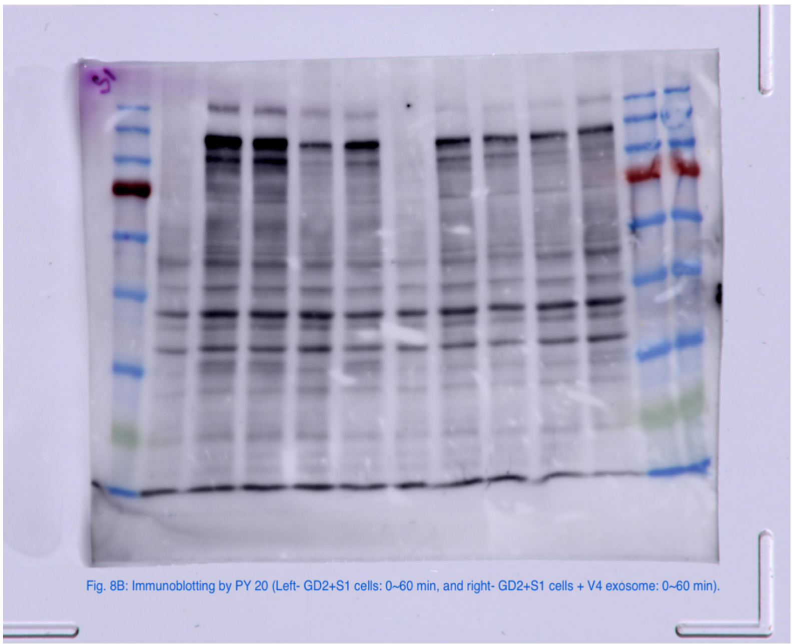

Fig. 8B: Immunoblotting by PY 20 (Left- GD2+S1 cells: 0~60 min, and right- GD2+S1 cells + V4 exosome: 0~60 min).

Fig. 8B: Beta actin (Left- GD2+S1 cells: 0~60 min, and right- GD2+S1 cells + V4 exosome: 0~60 min).

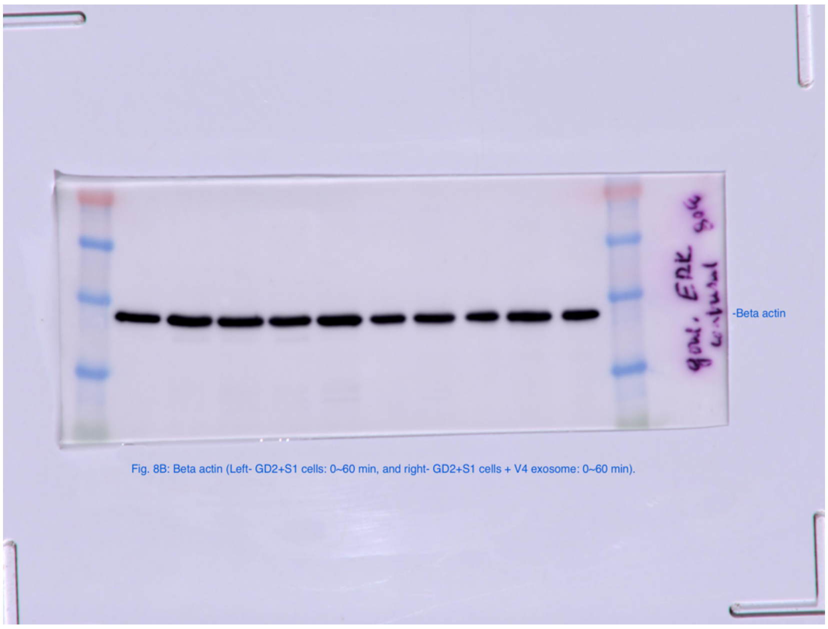

Supplement: Supplementary file 6 — Supplementary Information 6. [file 41598_2023_31216_MOESM6_ESM.pdf]
